# Mass spectrometry-based N-glycoproteomics for cancer biomarker discovery

**DOI:** 10.1186/1559-0275-11-18

**Published:** 2014-05-05

**Authors:** Ying Zhang, Jing Jiao, Pengyuan Yang, Haojie Lu

**Affiliations:** 1 Key Laboratory of Glycoconjuates Research Ministry of Public Health and Institutes of Biomedical Sciences, Fudan University Shanghai, 200032, China; 2 Department of Chemistry, Fudan University, Shanghai 200433, China

**Keywords:** Glycosylation, Disease proteomics, Mass spectrometry

## Abstract

Glycosylation is estimated to be found in over 50% of human proteins. Aberrant protein glycosylation and alteration of glycans are closely related to many diseases. More than half of the cancer biomarkers are glycosylated-proteins, and specific glycoforms of glycosylated-proteins may serve as biomarkers for either the early detection of disease or the evaluation of therapeutic efficacy for treatment of diseases. Glycoproteomics, therefore, becomes an emerging field that can make unique contributions to the discovery of biomarkers of cancers. The recent advances in mass spectrometry (MS)-based glycoproteomics, which can analyze thousands of glycosylated-proteins in a single experiment, have shown great promise for this purpose. Herein, we described the MS-based strategies that are available for glycoproteomics, and discussed the sensitivity and high throughput in both qualitative and quantitative manners. The discovery of glycosylated-proteins as biomarkers in some representative diseases by employing glycoproteomics was also summarized.

## Introduction

Proteomics, aiming at the large-scale protein analysis in biological samples, is useful for monitoring proteins that are involved in physiological changes in cells or organisms [1]. In the past two decades, proteomics has contributed tremendously to the discovery of biomarkers in a variety of diseases [2-5]. Although proteins perform a vast array of biological functions in living organisms, posttranslational modifications (PTMs) that are introduced after translation further extended the range of functions of the protein [3]. Glycosylation, one of the most common and functionally important PTMs in mammalian cells, plays a central role in many biological processes, including protein folding, host-pathogen interaction, immune response, and inflammation [4]. The most common types of protein glycosylations occur with the addition of specific glycan residues to asparagine (N-linked glycosylation). Alterations in either level or site of glycosylation can influence the cellular processes such as growth, differentiation, metastasis, and immune surveillance of tumors. Also, it is reported that modifications of the glycan structures are associated with several diseases as well. Since glycosylated-proteins have been discovered to be the clinical biomarkers for many diseases, for example, Her2/neu in breast cancer, prostate-specific antigen (PSA) in prostate cancer, CA125 in ovarian cancer, and carcinoembryonic antigen (CEA) in colorectal, bladder, breast, pancreatic and lung cancers, glycoproteomics, as a significant branch of proteomics, has become an emerging field for biomarker discovery [5,6]. In a long time, proteomics and glycomics are two separate disciplines with the proteomics studying the whole proteome (the protein), while the glycomics focusing on the glycome (the glycan). However, neither of them can provide complete information during the progression of diseases [7]. For instance, in some cases only the concentration of glycosylated-protein, the glycosylation occupancy on a certain site, or the structure of the oligosaccharide changed, while in most of the others, all three factors may vary at the same time. Therefore, it is necessary to have an inter-discipline of glycoproteomics that can not only qualify and quantify the proteins as the traditional proteomics, but also take the following two more aspects into account: 1) protein glycosylation sites (the occupancy extent of glycosylation sites); and 2) glycosylation forms (the alterations from the attached glycan compositions and structures). Glycoproteomics, as a combination of proteomics and glycomics, studying the profile of glycosylated proteins from both protein and glycan parts, would make unique contributions to the discovery of biomarkers of cancers.

**Figure 1:**
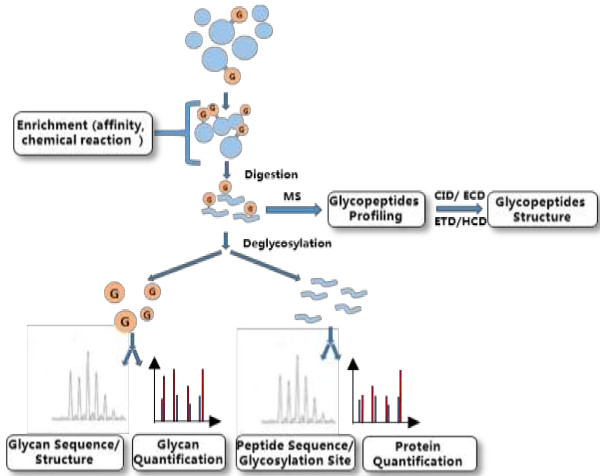
Strategy of the mass spectrometry-based N-glycoproteome.

Typically, there are several goals to be achieved in the development of potential biomarkers by glycoproteomics: 1) to identify the glycosylated-proteins, their glycosylation sites, and the glycan compositions; 2) to quantify the glycosylated-proteins and their glycosylation extent; 3) to validate the potential glycosylated-protein biomarkers; and 4) to establish the clinical test method. Complete glycoproteome analysis requires multiple methods, due to the complex linkage of the glycan to the protein and the variety of glycan compositions. In recent years, considerable works have been done to characterize the sequences of the glycosylated-proteins, the primary structures of the glycan attached to proteins, and the related glycosylation site occupancy. Mass Spectrometry analysis in combination with modern separation methodologies is one of the most powerful and versatile techniques used to study glycoproteome, because of its strong capabilities of providing both qualitative and quantitative information [8,9]. In general, N-glycoproteomics methodology consists of isolation and/or enrichment of glycosylated-proteins, proteolytic digestion, and detection of peptides for protein characterization and glycosylated-peptides for glycan composition analysis using MS (Figure 1). In N-linked glycosylated-proteins identification, the glycan are usually detached from the glycosylated-proteins/glycosylated-peptides using enzyme such as PNGase F [10]. Information of glycan composition, sequence, branching, glycosylation site, and relative quantification can thus all be obtained using MS-based methods. The conventional proteomics strategies have enabled enormous advances in the field during the past decade; however, they are still facing a bioanalytical challenge with the complex structure of glycans, which requires multiple MS-based approaches to gain different types of information.

In this review, we have summarized the current existing MS-based technologies that are utilized for characterization and quantification of N-glycosylated-proteins, and also the understanding of glycosylated-proteins as biomarkers of some representative diseases.

### Characterization of glycosylated-proteins by mass spectrometry

#### Glycosylated-proteins/ glycosylated-peptides enrichment

**Figure 2:**
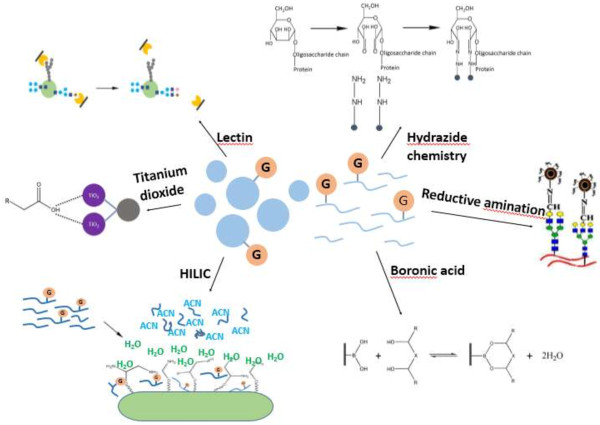
The common enrichment strategies for N-glycoprotein/glycopeptide.

A complete characterization of glycosylated-protein includes identification, glycosylated site location, glycan structure elucidation and the functional study of the glycosylated-proteins. However, the glycoproteome is overshadowed by the complicated cellular environment, the relatively low abundance (2% to 5%) and low ionization efficiency of glycosylated-peptides. Therefore, the effective separation of glycosylated-proteins/glycosylated-peptides combined with appropriate mass spectrometry fragmentation mode would facilitate more desirable identification result [1,11]. Generally speaking, the common enrichment method can be divided into affinity approach and chemical approach. The former is based on the interaction of sugar chains and stationary phase such as lectin affinity chromatography, titanium dioxide affinity chromatography and hydrophilic interaction liquid chromatography, while the latter realize immobilization of glycosylated-protein/glycosylated-peptide relying on the chemical reaction of glycans and specific functional group, such as hydrazide chemistry, reductive amination chemistry and boronic acid chemistry shown in Figure 2.

#### Lectin affinity chromatography

In the last decades, carbohydrate-binding proteins, known as lectins, have been widely exploited for the isolation of glycosylated-proteins or glycosylated-peptides from complex samples. Lectin has the capability to bind to distinct oligosaccharide epitopes. As early as 1970, Aspberg and Lloyd first applied lectin affinity for enrichment of glycosylated-proteins [12,13]. Since then, numerous research using lectins to enrich glycosylated-proteins/glycosylated-peptides have been developed. According to a fresh review, at least 160 lectins are known to researchers. Among them, more than 60 kinds are commercially available [14]. One kind of lectins includes concanavalin A (Con A) [15], wheat germ agglutinin (WGA) [16] and jacalin (JAC) [17], etc. They are all known to have broad specificity and can react with many different glycosylated-proteins. While, the more specific lectins, such as Sambucus nigra lectin (SNA), which is preferentially to sialic acid attached to terminal galactose in α-2,6 linkage, are often applied to adsorb a definite subset of the glycoproteome [14]. Therefore, the lectin method not only can target a broad type of glycans (e.g. Con A is often used for binding high-Man N-linked glycans) but also is appropriate for enrichment of glycans containing specific structures (e.g. L-phytohemagglutinin, or L-PHA, will recognize β (1,6)-branched N-linked glycans [18]) Lectin affinity chromatography based glycosylated-proteins/glycosylated-peptides enrichment strategy followed by downstream proteomic analysis has been successfully applied to variety kinds of biological samples, most frequently, in blood [19-23].

In the recent study of serum biomarker, Jung et al. took the advantage of narrow specificity lectins, which target more specific features of glycosylated-proteins to study disease associated aberrations in glycosylation [24]. They observed that approximately 30% of the plasma secretome can be captured using Con A alone, which has broad reactivity for the mannose cores typically found in N-linked glycans. Two fucose-specific lectins, Aleuria aurantia lectin (AAL) and Lens culinaris agglutinin (LCA), were found to bind 4% and 8% of the total plasma proteins, respectively. Highly specific Wisteria floribunda agglutinin (WFA)-assisted glycoproteomics approach was developed adopting dual enrichment. The combination of dissection of the WFA-stained cholangiocarcinoma tissue region and WFA-agarose column chromatography successfully identified 71 proteins as candidate markers with the assistance of subsequent analysis by mass spectrometry [23]. The limitation of enrichment approaches based on lectin is that no single lectin has the ability to bind the whole glycoproteome. Due to maximizing capture of majority of glycosylated-proteins in one step, enhanced binding strengths were often acquired by multi-lectin affinity chromatography. Kullolli et al. recently developed a High Performance Multi-Lectin Affinity Chromatography (HP-MLAC) composed of lectins ConA, JAC, and WGA. The effective integration of protein depletion, glycosylated-proteins enrichment and in-line sample concentration/desalting allowed the minimization of sample loss and the improvement of the depth of the plasma proteome analysis [25].

#### Titanium dioxide affinity chromatography

Titanium dioxide (TiO_2_) was often used to capture phosphopeptides from the complex sample. Larsen et al. presented a simple but highly selective strategy for enrichment of sialic acid-containing glycosylated-peptides [26]. The negatively charged sialic acid, which contains both carboxylic acid and hydroxyl group may interacts with TiO_2_ molecules via a multipoint binding. Therefore, after dephosphorylation by alkaline phosphatase, interaction will specifically occur between TiO_2_ and sialic acid. Under specific buffer conditions, nonglycosylated-peptides or neutral glycosylated-peptides were no longer bound onto the TiO_2_. A total of 192 and 97 sialic acid-containing glycosylation sites from depleted human plasma and saliva were identified, respectively in this way [26]. Zhao et al. made a profound profiling of the sialylated N-glycoproteomics in human plasma by an optimized strategy of combination of a filter-aided sample preparation, TiO_2_ chromatography enrichment, multiple enzyme digestion, and high-pH reversed-phase peptide separation. 982 glycosylation sites in 413 proteins were identified, providing the largest database in human plasma until now [27]. Considering that TiO2 displays high specificity to enrich the phosphopeptides, it can be utilized for comprehensive studies of both types of two PTMs simultaneously in one enrichment procedure [28].

#### Hydrophilic interaction liquid chromatography (HILIC)

Although hydrophilic interaction liquid chromatography (HILIC) was first introduced as early as 1970s to analyze saccharides [29], recent years have seen its versatile application for the separation of biomolecules. Relative to reversed phase liquid chromatography (RPLC), HILIC is featured by the use of a hydrophilic materials as stationary phase and a hydrophobic organic solvent as mobile phase, which can be regarded as a kind of normal-phase (NP) chromatography. HILIC makes it viable for the multidimensional separation of complex samples in proteomics applications, especially analysis of post-translational modifications. Yet, NP is often performed with non-water-miscible solvent buffers but the mobile phase used by HILIC is similar to those employed in the RP-LC mode. Therefore, HILIC has specific advantages such as less complicated eluent preparation, good solubility of polar samples and greater compatibility with electrospray MS. Richness of hydroxyls makes glycosylated-peptides more hydrophilic, which means that HILIC is suitable for glycosylated-peptide separation. Analytes and polar stationary phase are interacted with each other in the form of hydrogen bonding. However, the deep mechanism of HILIC is different because of various stationary phases.

Classical HILIC stationary phases were made of bare silica or silica gels modified with polar functional groups. For example, polyhydroxyethyl aspartamide column was used to help the enrichment of glycosylated-peptides from depleted plasma prior to MS analysis. Interestingly, the author demonstrated that TFA as an ion-pairing reagent could improve enrichment efficiency [30]. The idea of employing carbohydrate materials as modification of HILIC was based on hydrophilic binding principle of carbohydrate matrices such as cellulose and sepharose to oligosaccharides [31]. IgG isotype-specific Fc glycosylation profiles were characterized relying on HILIC-SPE with cellulose and sepharose stationary phases. The author pointed out sepharose-SPE displayed allowed a faster and more constant flow due to more homogeneous particle sizes [32]. Cotton wool, recently, was creatively introduced into HILIC SPE microtips to remove salts, most nonglycosylated peptides, and detergents. Purification of N-glycans after PNGase F deglycosylation treatment of IgG and transferrin was also obtained by cotton HILIC microtips [33].

Huang et al. put forward a new material bonded on HILIC silica with the help of diazotransfer reagent to transfer the partial amino groups in chitooligosaccharides to azido groups [34]. Compared to Sepharose, click-maltose on silica beads is more flexible, producing adequate hydroxyl groups to capture glycosylated-peptides and improving selectivity [35]. An automatical sample pretreatment workflow, composed of a click maltose-HILIC column for glycosylated-peptide enrichment, a strong cation exchange (SCX) precolumn for sample buffer exchange, and a PNGase F immobilized enzymatic reactor (IMER) for online deglycosylation was established, allowing improved sensitivity (detection limit of 5 fmol glycosylated-peptide) and selectivity (interfering 50 times (mass ratio) of BSA) [36].

The application of HILIC with zwitterionic materials is developing rapidly in the glycoproteomics. A charged liquid layer is generated near the stationary phase surface, creating a region of high ionic strength for polar interactions with glycan. The electrostatic interactions between the two oppositely charged groups is almost at a stoichiometric ratio, which is relatively weaker when compared to normal ionic exchangers such as SCX [37]. A two-dimensional chromatographic protocol with a combination of off-line ZIC-HILIC and RP chromatography including column packing, LC setup and gradient optimization was described. The strategy made it possible to improve sensitivity and handle small sample volumes, compatible with the stable isotopic label methods for quantitation used in glycoproteomics [38]. However, it is worthwhile to note that the selectivity of HILIC is relatively low as the coseparation of hydrophilic nonglycosylated-peptides.

#### Hydrazide chemistry

A solid-phase extraction method to enrich glycosylated-proteins based on hydrazide chemistry was first developed by Zhang et al. [39]. The methodology includes four steps: 1) Periodate oxidation of carbohydrate cis-diol groups to aldehydes. 2) Hydrazone formation between aldehydes and hydrazide groups on the support medium. 3) Tryptic digestion of the immobilized proteins in order to remove the nonglycosylated peptides. 4) Liberation of glycosylated-peptides from the support with PNGase F. Attributed to the good specificity and nice sensitivity of hydrazide chemistry-based enrichment approach, many kinds of complex biological samples have been investigated under this method. However, the glycan moiety was destroyed so the analysis of the structure of glycan was difficult. In order to make this isolation of glycosylated-protein/glycosylated-peptide easier, magnetic particles modified with hydrazide group may be synthesized [40-42]. Due to the enormous complexity of real biological samples, hydrazide chemistry combined with other pretreatment protocols will further increase the sensitivity and coverage of detection. For example, some pretreatments such as the depletion of high-abundant protein or separations by two-dimensional chromatographic will increase the sensitivity of the method [41]. Furthermore, increased glycosylated-protein sequence coverage was proved by a two-step protease digestion (Lys-C digestion, hydrazide resin separation, trypsin digetstion and PNGase F deglycosylation) [43]. Multiple enzyme digestion (trypsin, pepsin and thermolysin) significantly improved the coverage of N-glycosites [44]. In addition, Parker et al. adopted parallel enrichments strategy to reveal a deeper analysis of glycoproteome, with a total of 1556 nonredundant N-linked glycosylation sites from 972 protein groups identifications [45]. Compared with lectin method, hydrazide chemistry-based enrichment turned out to be more specific due to its covalent bonding to the carbohydrates.

#### Reductive amination chemistry

Recently, our group improved the traditional hydrazide chemistry by introducing reductive amination to enrich N-Linked glycosylated-peptides for the first time. The enrichment strategy is based on the reaction between aldehyde groups which were produced by oxidation of the cis-diol groups of glycans and amino groups modified on the magnetic nanoparticles. Compared to the hydrazide chemistry-based solid phase extraction, this protocol eliminated the desalting step and shortened the extraction time to 4 h, improving the detection limit of glycosylated-peptides by 2 orders of magnitude. A total of 111 N-glycosylation sites were identified in 108 glycosylated-peptides in only 5 μL of human serum [46].

#### Boronic acid chemistry

Boronate esters are selectively formed under alkaline condition and the reversed-reaction can occur under acidic conditions [47]. Therefore, boronic acid chemistry can be utilized for the capture and release of cis-diol-containing molecules such as glycans. The reversible, covalent bonds between boronic group and vicinal diols of glycans endow boronic acid “lectin-like” properties. However, the recognition between the glycosylated-peptide and boronic acid does not need a complicated discrimination motif, which means that boronic acid have a more broad binding ability to glycans than lectin alone. Besides, an easy switch of the pH will control the capture/liberation of glycoconjugates, which make boronic acid-based enrichment for glycosylated-proteins/ glycosylated-peptides attractive.

Our group took advantage of FDU-12 which showed good selectivity towards glycosylated-peptides to dramatically enhance the LOD of glycosylated-peptides by close to 2 orders of magnitude [48]. We further made use of mesoporous silica MCM-41 to be functionalized with boronic acid groups. The boronic acid-functionalized MCM-41 silica was demonstrated to have excellent selectivity (glycosylated-peptides/nonglycosylated-peptides at molar ratio of 1:100), good binding capacity (40 mg g^−1^) and high recovery of glycosylated-peptides (88.10%) and was applied to reveal rat serum glycopeptidome for the first time, identifying 15 unique glycosylation sites originating from 15 different endogenous glycosylated-peptides [49].

### Mass spectrometry analysis

Mass spectrometry is by far the most powerful analytical technique which allow the qualitative and quantitative analysis of glycosylated-proteins. Among the “modern” ionization methods, two methodologically distinct approaches to investigate the structures of glycoconjugates in complex biological sample with increasing effectiveness and measurement sensitivity are Matrix-Assisted Laser Desorption/Ionization (MALDI) and Electrospray Ionization (ESI). The heterogeneous characteristic of glycosylated-peptide complicates the corresponding MS analysis and therefore promotes different ionization experimental conditions for different samples. For example, neutral and basic glycosylated-peptides may be analyzed under positively charged ions mode, while acidic glycoconjugates (sialic acid and sulfate containing) tend to be detected more effectively as negatively charged [50].

A complete characterization of the structure of glycosylated-peptides should include not only determination of the peptide’s amino acid sequence, but also the confirmation the glycans and the site of glycosylation. The structure elucidation mainly relies on tandem MS analysis, through which important piece of data from the overall the glycosylated-peptide may be obtained. Therefore, an appropriate fragmentation method or a combination of multiple techniques is really necessary for a comprehensive analysis, which include both the information of glycan structure and glycosylation site. Collision-induced dissociation (CID) is one of the most widely applied fragmentation mode in which molecules are usually bombarded with an inert buffer gas to increase their internal vibrational energy and fragmentation occurs when enough energy is applied on the molecule. Therefore, in most cases, CID is not suitable for structural characterization because the obtained tandem mass spectrum is dominated by the glycans cleavages fragments. In order to generate maximum oxonium ion abundance and minimum peptide backbone fragmentation, Roepstorff et al. optimized the collision energies and realize more sensitive and selective glycopeptides identification [51].

A complementary fragmentation mode was electron-based methods, including electron-capture dissociation (ECD) and electron-transfer dissociation (ETD). Compared to CID, electron-based fragmentation pathways are prior to break the peptide bonds than glycans bonds, therefore more suitable for providing the reliable structural information about the glycosylation site because the modifications still remain onto the peptides. In ECD, low-energy electrons (<0.2 eV) were applied on multiply protonated peptide lead to the cleavage of N–Cα bonds to generate a series of c and z. fragments ions [52]. Similar to ECD, in ETD, the electron delivery is achieved through the singly charged anions (e.g., fluoranthene anions) to the multiply protonated peptides. The reactions can occur in ion traps, which are less expensive than the Fourier transform ion cyclotron resonance (FT-ICR) mass, in which ECD is performed. Both ECD and ETD results preferentially in c and z fragments ions. Therefore, ECD and ETD are two excellent tools for post translational modifications such as glycosylation and phosphorylation because the labile modifications still remain onto the peptide and are largely unaffected by the fragmentation process [53]. When used in combination with CID, the glycosylated-peptides may be characterized to the fullest. Four model glycosylated-proteins were used to evaluate the power of ETD in conjunction with CID in the characterization of tryptic glycosylated-peptides. Results showed that ETD have the ability to provide reliable structural information about the glycosylation site and when it was used in tandem with CID, and both the glycan structure and the amino acid sequence of the glycosylated-peptide could be deduced [54]. A systematic approach including enrichment, deglycosylation, mass spectrometric analysis with CID and ETD was established for the study of serum of hepatocellular carcinoma (HCC). By the comparison of matched spectra of glycopeptides with and without fucosyl residue, ETD can help to localize core fucosylation sites. The combination of the CID and ETD increased the coverage and confidence of fucosylation identification [55].

By coupling an linear ion trap (LTQ) to an orbitrap analyzer, the new type of mass spectrometer boasts significantly higher resolution much more improved mass accuracies than ordinary ion-trap [56]. Olsen et al. reasoned that the C-trap, which is originally used to store ions could be used as a collision chamber for fragmentation at higher energies [57]. This higher-energy collisional dissociation (HCD) was first used to characterize protein glycosylation by Segu et al. [58]. Distinct Y1 ions (peptide + GlcNAc) generated from HCD mode allowed location of N-glycosylation sites of glycosylated-proteins. Furthermore, fragment ions produced by HCD were detected in the orbitrap, overcoming 1/3 cut-off limitation. Therefore, the glycan oxonium ions can also be detected. Scott et al. enriched Campylobacter jejuni glycosylated-peptides by using ZIC-HILIC followed by CID/HCD as well as CID/ETD. Based on their respective advantages, CID/HCD allowed the identification of glycan structure and peptide amino acid sequence, while CID/ETD enabled the elucidation of glycosylation. 130 glycosylated-peptides and 75 glycosylation sites were identified among which CID/HCD provided the majority of the results (73 sites) [59]. Ye et al. adopted HCD as the supplemental dissociation technique for CID and ETD, which can generate complementary fragments for structural elucidation of the glycosylated-peptides. Five glycosylated-peptides were analyzed with 23 glycoforms identification and 100% protein sequence coverage [60]. In a word, despite the dissociation behaviors of glycosylated-peptide ions are complex, the diversity of dissociation pathways can be regarded as an opportunity to help us fully understand glycosylated-peptide fragmentation processes and lay foundation of glycosylated-peptide profiling [61,62].

### Quantification

**Figure 3:**
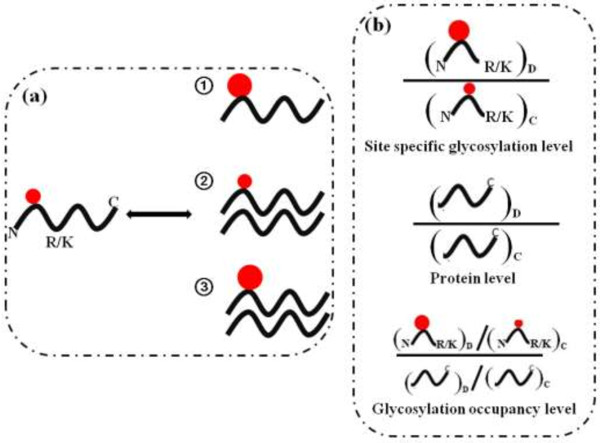
**The common quantitative aim and related strategies for N-glycoproteome. (a)** Interpreting alterations in glycoproteomics. ① Changes in site specific glycosylation level; ② Changes in protein expression level; ③ Changes in glycosylation occupancy level. Glycosylation site is depicted by a filled red circle. **(b)** Specific quantitative strategies for different goals. “D” denote disease sample, “C” denote control sample.

The high-throughput and accurate quantification of proteins is another essential component of proteomics strategies for studying cellular functions and processes. The progresses in quantitative proteomics have provided important insights into many biological processes and in discovering proteins as biomarkers. A number of robust and accurate MS-based quantitative methods have been developed for quantification of protein expression levels in large scale. Besides monitoring changes in protein expression levels as the traditional quantitative proteomics, quantitative glycoproteomic analysis should also determin the both the site specific glycosylation level and the glycosylation occupancy level. As shown in Figure 3 (a), a glycosylated-protein can change in three typical manner (taking upregulated in glycosylation level and/or upregulated in protein expression level as an example), ① Changes in glycosylation; ② Changes in protein expression level; ③ Changes in protein expression level with simultaneous changes in glycosylation level. In the whole proteome digests, the nonglycosylated-peptides come from both of the glycosylated-proteins and nonglycosylated-proteins, while the glycosylated-peptides are only from the glycosylated-proteins. Therefore, in case ①, the abundance of nonglycosylated-peptides keeps constant, while the abundance of glycosylated-peptides may change significantly; in case ②, the abundance of glycosylated-peptides remain unchanged, while the abundance of nonglycosylated-peptides may vary dramatically; in case ③, both the abundances of glycosylated-peptides and nonglycosylated-peptides change. According to the different purposes, specific quantitative strategies can be applied as shown in Figure 3 (b).

Different methods of quantitative glycoproteomics can be categorized into two groups: label-based and label-free. Label-based quantification is accomplished by calculating the ratio of mass spectrometric intensities between the “light” and “heavy” samples. These techniques are based on different mechanisms to introduce labeling tags on the peptides or proteins, including stable isotopic or isobaric labeling using chemical reactions (e.g. dimethylation and iTRAQ), metabolic incorporation (e.g. SILAC) and enzymatic reactions (e.g.^18^O labeling). While label-free quantification, which does not use a compound containing stable isotope to bind to the protein, is also a widely used technique for quantifying relative changes in complex protein samples.

### Relative quantification

#### Alteration at the site specific glycosylation level

The determination of the site specific glycosylation level usually combines glycosylated-proteins/glycosylated-peptides enrichment strategy, since separation of the glycosylated-proteins/ glycosylated-peptides from the backgrounds can reduce the sample complexity to a large extent. Quantification can be then performed by comparing the glycosylated-peptides with or without detaching glycan in different samples.

In most cases, quantifications are performed using peptides from glycosylated-peptides after detached glycan. Due to the multiple glycoforms that can be attached to the peptide backbone, glycosylated-peptides always have a high degree of heterogeneity, which contributes to the difficulties in glycosylated-peptides identification as well as quantification. Meantime, attachment of the glycans makes the mass spectrum difficult to decipher. Therefore, detaching the glycans from glycosylated-peptides is beneficial in the following aspects for quantification. First, when a glycosylated-peptide is identified in mass spectra, it would produce many spectral peaks corresponding to those different glycoforms attached to this glycosylated-peptides; the spreading of the glycosylated-peptide ion signal among all these glycoforms would lower the abundance of the MS signal of each peak. After detached the glycans, deglycosylated-peptides with the same amino acid composition would display as one peak in the mass spectra, making it easy to be elucidated.; Second, it is known that the deglycosylation step using peptide-N-glycosidase F (PNGase F) results in a conversion of asparagine to aspartic acid in the peptide sequence, introducing a mass difference of 0.9840 Da. The fixed mass difference combined with N-glycosylation sequence motif is conducive to further confirmation of the glycosylation site.

Once the glycans are detached, different glycoforms on the specific glycosylation sites would contribute to the total abundance of deglycosylated-peptides, the quantitative results would in fact reflect the total glycosylation profile on a specific glycosytion. Zhang et al. first described a method for the quantification of glycosylation profiles using deglycosylated-peptides after detaching glycans, for which, glycosylated-proteins were bound to a solid support using hydrazide chemistry, and labeled with isotopically light (d0, contains no deuteriums) or heavy (d4, contains four deuteriums) forms of succinic anhydride, followed by releasing the labeled deglycosylated-peptides via PNGase F. d0 and d4 labeled peptides were then sent to mass spectrometric analysis, and the relative MS intensity ratio between the two samples reflected the glycosylation profile. The robustness of the method has been demonstrated by the standard glycosylated-protein analysis, and the observed ratios were close to the expected, with the differences ranging between 0% and 29% with a mean of 8%. The authors then successfully applied this method in quantifying the glycoproteome of different samples, including cell membranes and human serum [39]. Glycosylation quantification can be completed with the combination of various enrichment methods and labeling methods. In Sun’s study, a solid phase based labeling approach by integration of glycosylated-peptides enrichment and stable isotope labeling on hydrazide beads was developed, with washing, labeling, and release of the glycosylated-peptides were all performed on the hydrazide beads sequentially. This approach was proved to be accurate and had a good linearity range with 2 orders of magnitude for quantification of deglycosylated-peptides. In addition, when comparing with dimethyl labeling conventionally performed in solution, the developed approach has better enrichment recovery (10 − 330% improvement) and high detection sensitivity. For example, when using only 10 μg of the four standard glycosylated-protein mixtures and 400 μg of bovine serum album interference as the starting sample, 42% of the annotated glycosites could still be quantified., higher than the convetional method of 26% [63]. Cordwell and co-workers quantified the glycoproteome of myocardial ischemia/reperfusion injury via iTRAQ labeling, and validated these changes with dimethyl labeling. The iTRAQ approach revealed 80/437 deglycosylated-peptides with altered abundance, while dimethyl labeling confirmed 46 of these and revealed an additional 62 significant changes. In their strategy, they employed a series of different enrichment methods including hydrazide capture, titanium dioxide and HILIC with multiple proteases to increase glycoproteome coverage [45]. Moreover, to enable the accurate and large scale quantification of the glycosylated-protein, Mann and co-workers developed a method combined “filter aided sample preparation” (FASP) and super-SILAC to quantify N-glycosylated secretome during breast cancer progression and in human blood samples. The N-glycoproteome from the 11 cell lines that are representative of different stages of breast cancer development were obtained by the FASP flow and then analyzed by LC-MS/MS. Then a super-SILAC mix was developed by collecting conditioned medium from three representative different heavy stable isotope labeled cell lines, which served as an internal spike-in standard when being mixed with the conditioned medium of the tested cell lines for all samples accurate quantification. A total of 1398 unique N-glycosylation sites were identified and quantified, and biologically relevant differences were clearly reflected by N-glycosylated secretome profiles [64].

If the enrichment strategy is performed at the protein level, nonglycosylated-peptides from the glycosylated-proteins can also be used to quantify the glycosylation profile. As glycosylations only occur on a less part of the amino acid, it is obvious that the nonglycosylated-peptides are dominated in the whole proteome digest. If all of the non glycosylated-peptides are derived from glycosylated-proteins, the nonglycosylated-peptides can be used as the substitutes of glycosylated-peptides for quantification the total glycosylation profile, which would make the quantification more accurate and reliable due to more peptides being employed. Zou et al. developed a relative quantitative method for profiling the glycoproteome of human serum from hepatocellular carcinoma patient and healthy people, using nonglycosylated-peptides derived from glycosylated-proteins instead of glycosylated-peptides. They first captured glycosylated-proteins from serum samples on a solid-phase to ensure that the nonglycosylated-proteins would not be retained for further analysis. Therefore, all of the nonglycosylated-peptides came from the glycosylated-proteins. Then, nonglycosylated-peptides were released from the solid-phase of by trypsin digestion, separated by two-dimensional liquid chromatography, and analyzed by tandem MS. Protein quantification was achieved by a label-free quantitative method, which compared the spectrum counts of identified nonglycosylated-peptides between different samples. This method was demonstrated to have almost the same specificity and sensitivity in glycosylated-proteins quantification as capture at glycosylated-peptides level. The differential abundance of proteins present were quantified with high confidence at the concentration as low as nanogram per milliliter levels [65]. For the accurate and specific quantification of the cell surface glycosylation profile, they further proposed a modified cell surface-capturing strategy, where the glycosylated-peptides were submitted to LC-MS/MS analysis directly for identification of glycosylated-proteins, and the nonglycosylated-peptides were isotopically labeled for quantification of glycosylated-proteins. 33 glycosylated-proteins were quantified with significant expression change between the two cell lines of Chang Liver and HepG2 cells [66].

While most of the quantitation methods were based on deglycosylated-peptides, Ye and co-authors reported a quantification method achieved by comparing glycosylated-peptides without detaching the glycans in a recent study, combining a comprehensive LC-MS analysis method with tandem mass tag (TMT) labeling. Because the glycans are retained, the quantitative results are closely related to the glycoforms, it is a glycoform specific glycosylation quantitation. The glycosylated-peptides were directly qualitatively and quantitatively characterized on an Orbitrap mass analyzer with data-dependent tandem mass spectrometry (MS/MS) by alternating collision-induced dissociation (CID), electron-transfer dissociation (ETD), and higher-energy collisional dissociation (HCD) scans. The CID and low-energy HCD MS/MS analysis were performed to generate fragment ions by cleavage of glycosidic bonds, and these fragments were used for peptides sequencing and glycan structure elucidation. In addition, isomeric glycosylated-peptides with a slightly different sequence of glycan moiety could also be differentiated by CID. ETD, which tended to preserve labile PTMs, allowed not only the identification of the amino acid sequence of a glycosylated-peptide but also the assignment of its glycosylation sites. HCD combined with a TriVersa NanoMate that used to collect and reinfuse target glycosylated-peptides fractions, were performed to generate reporter ions from the tandem mass tag, and thus, provided the quantitative information for glycosylated-peptides. By alternatively using three different dissociation methods, 23 glycoforms from all 5 corresponding glycosylated-peptides were identified from tryptic digests of bovine fetuin. Nonglycosylated-peptides and glycosylated-peptides were simultaneously quantified with quantification errors less than 9% for all examined peptides and the results from nonglycosylated-peptides and glycosylated-peptides were comparable with % RSD less than 10 in most cases. However, because of the complexity of real sample, this method has only been applied to the standard sample analysis [60]. Radoslav et al. reported a LC-MS-multiple reaction monitoring (MRM) workflow that using glycosylated-peptides to quantify the glycan-specific glycosylation of Haptoglobin in liver cirrhosis and hepatocellular carcinoma. To achieve quantification of the Hp-T3 glycoforms, intense oxonium ions that are easily generated in CID of glycosylated-peptides, were selected for quantification. The glycoform-specific oxonium and peptide-HexNAc fragments were used to assure specificity of detection. The quantitative analysis showed that 14 multiply fucosylated glycoforms increased significantly in the liver disease group, comparing to healthy controls with an average 5-fold increase in intensity (p < 0.05). At the same time, 2 tri-antennary glycoforms without fucoses did not increase in the liver disease group and 2 tetra-antennary glycoforms without fucoses showed a marginal 40% increase in intensity at most. However, the complex glycosylated-peptide mixtures made it difficult to realize the specificity of these transitions, therefore, this strategy should begin with the purification of target glycosylated-protein from patient plasma [67].

#### Alteration at the protein level

Quantitation of the alteration at the protein level is quite similar to the traditional quantitative proteomics analysis and it can be performed through label-based or label-free approaches. In these cases, enrichment strategy can be omitted because the quantification focuses on the total amount of proteins including their native states and glycosylated counterparts. Glycosylation site is only qualitative analysis by mass spectrometry to affirm the glycosylated-protein. Because the whole procedure can be found in traditional quantitative proteomics, we will not discuss in detail in this review.

#### Alteration at the glycosylation occupancy level

Glycosylated site occupancy is defined as the percentage of the protein that a site is occupied by glycan. So when analyzing the alteration in the glycosylation occupancy level, the alteration of glycosylation extent and the alteration of protein should be both taken into consideration. Because nonglycosylated-peptides can be from either glycosylated-protein and or their glycosylated-protein counterparts, the total nonglycosylated-peptides of a protein reflect the level of this protein including its native state and glycosylation state. While to obtain the glycosylation profile, glycosylated-proteins are always first digested into peptides, among which, the glycosylated-peptides are then selectively enriched to reflect the glycosylation profile. The glycosylation site occupancy, then, can be calculated from the ratio between the glycosylated-peptides and nonglycosylated-peptides (including ones that are from non glycosylated-proteins and its glycosylated counterparts). Our group has developed a tandem ^18^O stable isotope labeling (TOSIL) method to quantify the changes in glycosylated-proteins expression and the individual N-glycosylation site occupancy. Digests of two samples for comparison were labeled in H_2_^18^O or H_2_^16^O catalyzed by trypsin to introduce a mass tag on the C-termini of peptides, and the glycans were then released with PNGase F hydrolysis in H_2_^18^O or H_2_^16^O respectively to introduce a mass tag of ^18^O or ^16^O on the glycosylation site. As a result, the relative quantities of total protein level were obtained by measuring the ^18^O/^16^O intensity ratios of nonglycosylated-peptides, while the relative quantities of glycosylated-protein level could be obtained by measuring the intensity of deglycosylated-peptides. Therefore, N-glycosylated site occupancy could be obtained by comparing the ratio of glycosylated-proteins level and total protein level of the two samples. By employing the TOSIL approach, it yielded a good linearity for the quantitative response of the N-glycosylated site occupancy, which was within a 10-fold dynamic range with a correlation coefficient r^2^ > 0.99. This strategy was then successfully applied to the analysis of serum glycoproteome between ovarian cancer patients and healthy controls, and results showed that changes in N-glycosylation occupancy at specific sites did not always have the same trends as those of protein expression levels [68]. In addition, Shakey et al. developed a similar strategy with the same labeling methods but different enrichment method for relative quantitation of glycosylated-proteins site occupancy [42]. Ueda et al. reported an approach termed isotopic glycosidase elution and labeling on lectin column chromatography (IGEL) for profiling serum biomarkers in lung cancer patient serum. Protein digests were first labeled with 8-plex iTRAQ reagent, followed by the capturing of glycosylated-peptides with lectin-column chromatography and the tagging of the N-glycosylation sites with ^18^O by PNGase F. Nonglycosylated-peptides labeled with iTRAQ were used to quantify the glycosylated-proteins in the protein expression level, whereas deglycosylated-peptides labeled with iTRAQ were used to quantify the glycosylation degree. With the aid of a 8-plex labeling reagent, this technology allowed the comparisons of up to eight different samples within a single experiment [69].

### Absolute quantification

Quite often, the “hypothesis-generating” relative quantification experiments mentioned above are worthless for defining the abundance of a protein. Therefore, subsequent experiments are strongly needed to target them explicitly through absolute quantification. Targeted quantification of proteins is a daily task in biological research, but traditional immunoaffinity-based methods cannot reach desired levels of sensitivity and throughput. In conjunction with the stable isotope dilution method, MS-based selected reaction monitoring (SRM) and MRM assays have opened new avenues for absolute quantification of the target protein, with unparalleled sensitivity and high-speed [70,71]. Domon et al. designed a heavy isotope–labeled synthetic peptide with C-terminal lysine and arginine labeled with ^13^C and ^15^ N, respectively. Especially, the asparagine (N) at the N-X-S/T motifs was replaced with aspartic acid (D) to mimic the deglycosylated-peptides. Then the heavily labeled peptides were used in the SRM workflow for absolute quantitative of the target glycosylated-proteins. However, this protocol can only quantify the concentration at the protein level, while the degree of glycosylation could not be distinguished [72]. In recent work, Ruedi et al. reported a large scale absolute quantitative method for N-glycoprotome by SWATH-MS. SWATH-MS is a data-independent (DIA) acquisition mass spectrometric method in which data are acquired on a fast, high resolution Q-TOF instrument. By repeatedly cycling through sequential isolation windows over the whole chromatographic elution range, SWATH generates a complete digital archive of all possible ions in a single analysis for permanent, retrospective, quantitative MS/MS analysis of complex samples. It thus can thus potentially combine the advantages of shotgun (high throughput) with those of SRM (high reproducibility and sensitivity). The author utilized isotopically labeled heavy forms (containing either a C-terminal [^13^C6^15^N4] Arg or [^13^C6^15^N2] Lys residue) of deglycosylated-peptides as the “absolute quantification” (AQUA) peptides to realize the quantification. Their results showed that compared to SRM, SWATH acquisition allowed the quantification of plasma glycosylated-proteins with equal variances, similar accuracy, and dynamic range, except for a slightly higher LOQ (2–3 times less sensitive) [73]. While the above-mentioned works focused on the absolute quantification of glycosylated-proteins, Ruhaak et al. have developed a method using MRM to examine quantitative changes in glycosylation at a site-specific level. They first performed studies on tuning the MS conditions for nonglycosylated-peptides and glycosylated-peptides, using standard IgG to construct suitable transitions. Then, the absolute concentrations of proteins were obtained using nonglycosylated-peptides and absolute glycosylated-proteins concentration and site-specific quantitation of individual glycoforms were obtained using glycosylated-peptides. Both nonglycosylated-peptides and glycosylated-peptides were quantified using MRM in the same run. Consequently, this new approach provided information regarding both the absolute amount of protein and the site-specific glycosylation profile, which is useful to determine if altered glycosylation profiles in serum/plasma were due to a change in protein glycosylation or a change in protein concentration. This strategy also showed a low limit of detection of 60 attomoles, and a wide dynamic range of 3 orders magnitude for IgG protein quantitation [74].

### Diseases

N-linked glycosylated-proteins, have been shown to be increasingly important in biomarker analyses and biopharmaceutical drug development [75]. Aberrant protein glycosylation may result in abnormal changes in biological function/activity, protein folding, and molecular recognition in cancer. The site of protein glycosylation and the structure of the oligosaccharide could also be altered during initiation or progression of disease [75,76]. Cancer has emerged as the most dreadful disease in the world. Although there has been a great deal of progress in treating it in the last decades, cancer still claims hundreds of thousands of lives each year. The discovery of these cancer-associated modifications of glycans on the glycosylated-proteins may also improve on the specificity of existing cancer biomarkers. For example, elevation of serum alpha-fetoprotein (AFP), a common marker for hepatocellular carcinoma (HCC), also occurs in non-HCC conditions such as pregnancy. In contrast, AFP-L3, consisting of core-fucosylated glycoforms of AFP, provides better specificity for HCC. The most deadly forms of cancer in the world include lung cancer, colon cancer, breast cancer, pancreatic cancer, liver cancer et al. We will discuss recent advances of glycoproteomics in these common cancers in the following parts to demonstrate the usefulness of glycoproteomics in biomarker discovery, diagnosis and subtype classification et al.

Breast cancer is the most frequently diagnosed cancer, and it is the leading cause of cancer death among females. Recent research reported by Yen et al. showed that glycosylated-proteins could be used to distinguish various subtypes of breast cancer, as well as to distinguish normal and benign breast cells from breast cancer cells. They obtained glycosylated-protein profiles, characterized these glycosylated-proteins from 14 breast cell lines, and then sent the glycoproteome to unsupervised hierarchical cluster analysis. Results showed that the differential expression of glycosylated-proteins in these breast cancer cell lines readily allows the classification of the lines into normal, benign, malignant, basal, and luminal groups [77]. Lung cancer is the leading cancer in males. Through quantification using MS-based glycoproteomic approaches, Tsai and co-authors noticed that when compared to normal donors, the fucosylated haptoglobin (Hp) level increased significantly in serum of each subtype of lung cancer. They also determined the glycan structure to be an alpha 2,6-linked tri-sialylated trian-tennary glycan, containing alpha 1,3-linked fucose which is attached to the four-linked position of the three-arm mannose of N-linked core pentasaccharide [78]. As hepatocellular carcinoma (HCC) is the most common primary malignant tumor of the liver, Liu et al. developed an integrated platform to discover the glycosylated-protein biomarkers in early HCC. The approach they employed is to use lectin arrays combined with LC-MS/MS-based quantitative glycoproteomics. Lectin arrays analysis showed an elevation of fucosylation level of serum glycosylated-proteins from HCC compared with cirrhosis patients. Finally, C3, CE, HRG, CD14 and HGF were found to be biomarker candidates for distinguishing early HCC from cirrhosis, with a sensitivity of 72% and specificity of 79% [79]. Among the newly discovered potential biomarkers in HCC, AFP-L3 (a glycoform of alpha-fetoprotein, AFP) is recently approved by FDA as as a supplemental test in patients with elevated total AFP. Because elevation of serum AFP, a common marker for hepatocellular carcinoma (HCC), also occurs in non-HCC conditions such as pregnancy, AFP-L3, consisting of core-fucosylated glycoforms of AFP, provides better specificity for HCC. AFP-L3 levels have been found to be related to progression from moderately differentiated to poorly differentiated tumors, therefore, it may be used as an early diagnosis of HCC when the tumor diameter is <2 cm [80]. Similarly, in order to discover potential glycosylated-proteins biomarkers in ovarian cancer, Wu et al. applied a lectin array and Exactag labeling based quantitative glycoproteomics approach. After being labeled with isobaric chemical tags, glycosylated-proteins were enriched by fucose-specific lectin array. The enriched glycosylated-proteins were then identified and quantified by LC-MS/MS, and five glycosylated-proteins were found to be differentially expressed in the serum of ovarian cancer patients, compared to benign diseases. With the further confirmation by lectin-ELISA and ELISA assay, corticosteroid-binding globulin (CBG) and other four glycosylated-proteins were identified as potential markers for differentiating ovarian cancer from benign diseases or healthy controls. Especially, the combination of CBG, SAP, and CA125 showed improved performance for distinguishing stage III ovarian cancer from benign diseases compared to CA125 alone [81]. These above findings clearly suggest that glycosylated-protein modifications may be a useful means to identify novel markers for detection of cancer and monitoring of cancer progression.

## Conclusions

Despite intense activities in the field of biomarker research, new biomarkers are still urgently needed to accelerate efforts in developing new drugs and treatments of known diseases. Though the field of glycoproteomics is moving forward, and more glycosylated-protein biomarkers have been discovered, there is still much work to do, for the future glycoproteomics research. First, since biologically significant glycosylated-proteins often exist in low abundance, enrichment of these glycosylated-proteins with high specificity is still worth attention. Second, although traditional proteomics identification strategies can provide information on novel sites of glycosylation, software and bioinformatics methods still need to be developed to improve glycan composition and structural analysis. Third, in order to realize effective biomarker screening, it is required to accurately quantify the glycosylation site occupancy from both relative and absolute aspects, as well as to analyze the relationship between the changes in glycosylation site occupancy and protein expression. With the progress of glycoproteomics research, we believe that the growth in the development of methods for high-throughput glycoproteomics will shed new lights on the discovery of new biomarkers.

## Competing interests

The authors declare that they have no competing interests.

## Authors’ contributions

YZ and JJ drafted the manuscript. All authors edited and approved the final manuscript.
